# Intensive Lifestyle Intervention for Cardiometabolic Prevention Implemented in Healthcare: Higher Risk Predicts Premature Dropout

**DOI:** 10.1177/15598276241259961

**Published:** 2024-06-19

**Authors:** Benno Krachler, Anna Söderholm, Fanny Ekman, Frida Lindberg, Joakim Lindbäck, Johan Nilsson Sommar, Eva-Lotta Glader, Bernt Lindahl

**Affiliations:** 1 Department of Public Health and Clinical Medicine, Sustainable Health, 59588 Umeå University, Umeå, Sweden (BK, AS, FE, FL, JL, JNS, EG, BL); 2 Region Västernorrland, Livsstilsmedicin Österåsen, Sollefteå, Sweden (BK); 3 Department of Psychology, Umeå University, Umeå, Sweden (AS)

**Keywords:** treatment adherence, health behavior, lifestyle risk reduction, early medical intervention, primary prevention, preventive health programs

## Abstract

**Aims:**

Patient characteristics and treatment setting are potential predictors of premature dropout from lifestyle interventions, but their relative importance is unknown.

**Methods:**

From the quality registry of the unit for behavioral medicine, Umeå University hospital, we identified 2589 patients who had been enrolled in a multimodal lifestyle intervention for cardiometabolic risk reduction between 2006 and 2015. Baseline characteristics predicting dropout before 1-year follow-up were selected by a stepwise logistic regression algorithm.

**Results:**

Better physical health and older age predicted full participation, with odds ratios for premature dropout (ORs) of .44 (95% confidence interval (CI) .31-.63), and .47 (95% CI .34-.65) in the highest compared to the lowest quartile, respectively. Odds of premature dropout were also lower among female participants, .71 (95% CI .58-.89). Premature dropout was predicted by higher BMI, snuffing tobacco, and smoking, with ORs of 1.53 (95% CI 1.13-2.08) in the highest compared to the lowest quartile of BMI, 1.37 (95% CI 1.03-1.81) comparing snuff user with non-users and 2.53 (95% CI 1.79-3.61) comparing smokers with non-smokers. Odds ratio for premature dropout among inpatients compared with outpatients was .84 (95% CI .68-1.04).

**Conclusion:**

Higher risk at baseline predicts premature dropout.

“For women and tobacco-users, inpatient-setting was associated with higher rates of full participation compared to outpatient-setting.”

## Introduction

Non-communicable diseases—mainly cardiovascular disease, diabetes, chronic respiratory disease, and cancer—are closely related to lifestyle and are therefore—at least in part—preventable by targeting everyday behavior.^1-3^ Lifestyle modifying treatment programs are efficient in both primary and secondary prevention, although efficacy is dependent on treatment adherence.^4-6^ Non-adherence is a multifactorial problem occurring in most types of treatments and is affected by factors related to socio-economic circumstances, the health care system, the patient’s general health condition, as well as program type and setting.^7-9^ Improving adherence in pharmacologically oriented secondary cardiovascular prevention is considered to be challenging.^10^ That challenge may be even bigger in multimodal lifestyle-oriented primary prevention where patients are required to reconsider not only adherence to medication, but several habits of everyday life—often in the absence of symptoms.^6,11^ Full participation in an intervention is an important component of adherence and a prerequisite of intervention-induced behavior change.^12^

Based on data from an ongoing treatment program implemented in public health care, the primary objective of this study is to identify patient characteristics that predict full participation/premature dropout from the intervention. As similar intervention-content is offered in both in- and outpatient setting, treatment setting is one of the few variables that can be affected easily by the care provider. Therefore, our secondary objective is to identify patient characteristics at baseline that predict lower rates of dropout in a particular setting. Identification of possible predictive baseline characteristics might enable us to adapt and personalize the program to reduce risk for premature dropout.

Based on findings from a previous internal quality-review our hypotheses were (i) patients who drop out prematurely from the intervention have baseline characteristics that differ from those who participate fully (i.e., including 1-year follow-up) and (ii) patients with low self-reported physical and mental well-being at baseline have higher rates of full participation in the program when treated in inpatient-setting.

## Methods

### Study Population

The study is based on data from the patient-registry of the department of Behavioral Medicine, Umeå University Hospital. Umeå is the capital of Västerbotten, the largest county of Northern Sweden with 270000 inhabitants. The lifestyle intervention is part of ordinary, government-financed health care. Patients are mainly referred from primary care physicians or lifestyle-nurses connected to the Västerbotten Intervention Program.^13^ To qualify for the treatment, patients had to have elevated risk for cardiovascular disease and/or diabetes. In addition to overweight/obesity at least one other metabolic risk factor, such as dyslipidemia, hypertension, or prediabetes was required. In an initial assessment and examination by a medical doctor, contraindications (uninvestigated symptoms of disease, inability to participate in group sessions, untreated alcohol- or drug addiction) were ruled out. All participants were 18 years or older. For the present study, we used data collected at baseline and 1-year follow-up. Data were included from 3106 patients who started participation in the multimodal lifestyle intervention program between 2006 and 2015: 1963 patients had been treated in the inpatient-setting at Sorsele wellness center, in the North of the county and 1143 in the outpatient-setting at Umeå. Decision on participation in in- or outpatient setting was mostly based on geography: Patients living within commuting distance of the outpatient clinic in Umeå, were generally offered the outpatient program whereas all others were generally assigned to the inpatient-setting. Exceptions were made, for the rare cases where patients expressed preference for either setting.

### The Intensive Intervention Program

**Figure 1.**
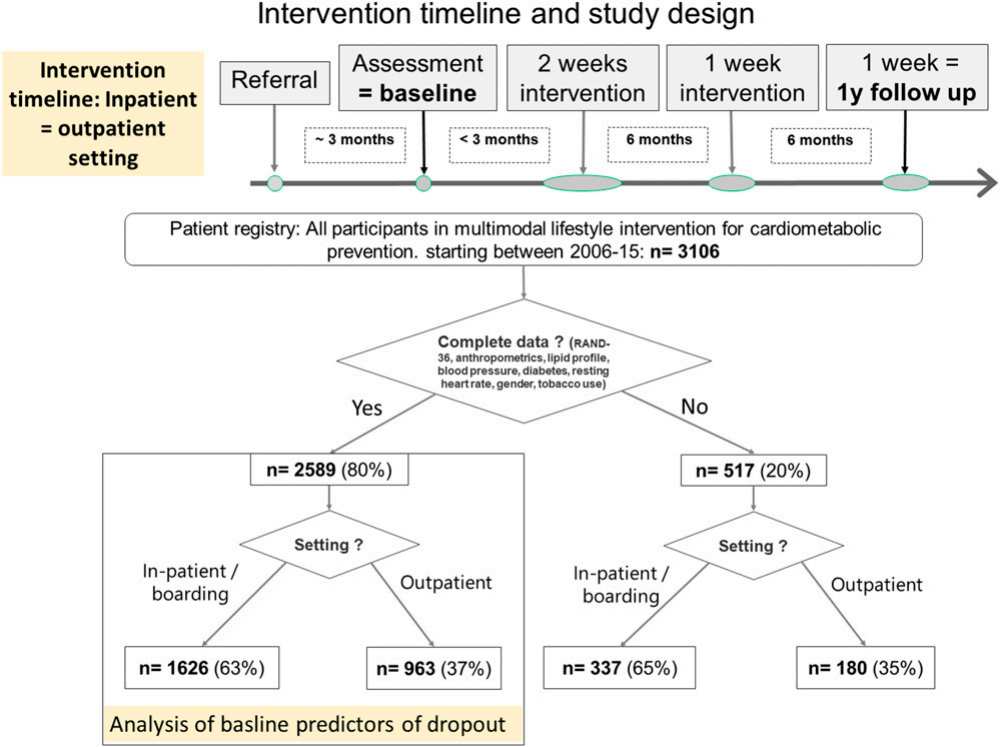
Intervention timeline and study design.

Study design and timeline for the intervention program in the period of data collection are given in Figure 1. An initial 2-week session was followed by another week 6 months later and a final week one year from baseline (1-y follow-up). Patients who were unable to attend any of the sessions were offered participation at a later date. Intervention design and content was similar for both in- and outpatient setting and has only changed slightly between its original conception in the 1980s and 2017. Both content and patient experience of the intervention program have been described, before.^14,15^ Briefly, aerobic activity of moderate intensity (e.g., brisk walks, gymnastics, bicycling and swimming) aiming at 60% of age-adjusted peak heart rate were performed daily for 2.5 h. A diet following the (then) current version of Nordic nutrition recommendations^16^ with a particular emphasis on vegetables was served. Portion sizes were calculated to give a daily energy intake of 7.6 MJ for men and 6.3 MJ for women. Behavioral elements of the intervention were group-based, that is, groups of 5-9 patients had sessions with a staff member trained in group-based motivational interviewing. Informal interaction both among patients and between patients and staff reinforced the message from lectures and group sessions. Intake of alcohol was prohibited at the treatment facilities and all tobacco-users were offered a cessation intervention. In total, patients were exposed to 140 h of behavior change techniques. Following the classification by Michie et al, behavioral practice/rehearsal (8.1), instruction on how to perform the behavior (4.1), demonstration of the behavior (6.1) and reduction of negative emotions (11.2) are the most frequently used techniques during the intervention.^17^ Social support (3.1), goal setting (1.1, 1.3), self-monitoring (2.3, 2.4, 2.5), and problem-solving (1.2) were encouraged for use at home. Exposure time for the various behavior change techniques for a similar intervention—derived from the one of the current study—was described, recently.^18^

### Measures

From the clinics’ quality registry, we retrieved variables on age, sex, anthropometrics, blood pressure, resting heart rate, lipid profile, fasting glucose, tobacco habits and heredity for diabetes. Health-Related Quality of Life (HRQoL) was assessed by RAND-36. Aspects of physical and mental well-being were considered and measured in terms of MH-Mental Health, RE-Role functioning Emotional, SF-Social Functioning, VT-vitality, GH-General Health, BP-Bodily Pain, RP-Role functioning Physical and PF-Physical Functioning. Low scores indicate worse overall health.^19^ For the current study we calculated composite scores as unweighted linear combinations of subscale scores for physical (GH, BP, RP, PF) and mental (MH, RE, SF, VT) health. This approach has recently been demonstrated to yield satisfactory criterion and convergent validity in a Scandinavian population.^20^ Reference data for RAND-36 were obtained from the Mid-Swed Health Survey.^21^

### Definition of Non-Adherence

In the context of a lifestyle intervention adherence has two aspects: First, full participation in the intervention, that is, attending all sessions of the program, and second implementing lifestyle-changes regarding food, exercise, stress management and other relevant habits of daily living. This study is only concerned with the first aspect of adherence, that is, attending all sessions of the program including 1-y follow-up. For clarity, the terms *full participation* and *premature dropout* are used to characterize adherence and non-adherence, respectively.

### Statistical Analysis

To limit loss of statistical power due to incomplete data, only variables with a response rate of ≥85 % were included in these further analyses. For all continuous variables, participants were classified into quartiles, cutoff values are given in appendix table 1. To identify baseline predictors of premature dropout, all variables were used in a stepwise multiple logistic regression analysis (SAS procedure PROC LOGISTIC) with a stepwise forward selection algorithm that starts with just the intercept and then sequentially adds the effect/variable that most improves the fit. Significance level for entry into the model was set at .3. The process terminates when no significant improvement can be obtained by either adding or removing any effect/variable. Patients with missing data regarding any of the chosen variables are excluded from further analysis. Iteration data for the adjusted model is provided in appendix table 2. To determine baseline characteristics associated with lower rates of premature dropout in a particular setting, treatment setting was included as interaction term in an additional analysis. As there was significant interaction with four different variables, analyzing all interactions in the same model would have resulted in a large number of different strata, precluding meaningful interpretation. We therefore built four different models, illustrating interaction of setting with the above-mentioned variables, separately. Iteration data for these models is provided in appendix table 3. All analyses were conducted using the Statistical Analysis System (SAS) 9.4 (SAS Institute Inc, Cary, NC, USA).

### Ethical Considerations

As poor living habits are more common among underprivileged parts of the population, optimizing lifestyle interventions meets not only the ethical principles of autonomy and beneficence, but also that of justice. Ethical approval for processing personal health data from Behavioral Medicine’s registry was issued by the Regional Board of Ethical Approval, Umeå, Nr 2018/224-31. The research plan was reviewed by the GDPR manager of Region Västerbotten prior to granting access to the registry data.

## Results

Background characteristics of participants with complete data are shown in Table 1. A comparison with those 20% who were excluded due to incomplete data is given in appendix table 4. Patients treated in the outpatient-setting were younger, had higher BMI, higher blood pressure, lower fasting glucose, a more favorable lipid profile, both higher role-and physical function scores. However, these differences were small (0,1-0,5 SD). Dropout rate was 21% for the outpatient—and 17% for inpatient-setting.

**Table 1.** Intensive Lifestyle Intervention Program: All Participants, Baseline Data.

Inpatient-setting n= 1626						Outpatient setting n= 963					
Mean	95% CI			SD	Continuous variables	Mean	95% CI			SD	P_in = outpat_
48.6	47.9	—	49.3	11.26	Age (years) ***	52.4	51.8	—	52.9	10.53	**.0000**
107.1	105.8	—	108.4	20.51	Weight (kg) ***	102.3	101.3	—	103.3	20.52	**.0000**
1.7	1.7	—	1.7	.09	Height (cm) *	1.7	1.7	—	1.7	.09	**.0115**
36.7	36.4	—	37.1	5.58	BMI (kg/m^2^) ***	35.5	35.2	—	35.8	5.96	**.0000**
115.7	114.8	—	116.5	13.32	Waist circumference (cm) ***	113.5	112.9	—	114.2	14.12	**.0001**
137.7	136.6	—	138.7	16.68	Systolic blood pressure (mmHg) ***	129.2	128.5	—	129.9	15.08	**.0000**
85.0	84.3	—	85.6	10.72	Diastolic blood pressure (mmHg) ***	83.3	82.9	—	83.8	9.74	**.0001**
75.6	74.9	—	76.4	12.06	Resting heart rate (beats/min)	75.6	74.9	—	76.4	13.06	.9836
6.0	5.9	—	6.1	1.77	Fasting glucose (mmol/L) ***	6.3	6.2	—	6.4	2.23	**.0001**
5.3	5.2	—	5.3	1.09	Cholesterol (mmol/L) **	5.4	5.3	—	5.4	1.15	**.0367**
1.3	1.2	—	1.3	.34	HDL (mmol/L) **	1.2	1.2	—	1.2	.37	**.0049**
3.2	3.2	—	3.3	.96	LDL (mmol/L)	3.3	3.2	—	3.3	1.03	.2316
1.8	1.7	—	1.8	.93	Triglycerides (mmol/L) ***	2.0	1.9	—	2.1	1.33	**.0000**
RAND-36 dimensions:											
71.9	70.6	—	73.2	19.85	Physical functioning (PF score) ***	68.1	67.1	—	69.2	21.59	**.0000**
56.8	54.3	—	59.2	38.95	Role functioning Physical (RP score) ***	49.5	47.5	—	51.5	40.59	**.0000**
55.9	54.2	—	57.5	26.22	Bodily pain (BP score) *	53.2	52.0	—	54.5	26.11	**.0136**
47.2	45.9	—	48.5	20.02	General health (GH score)	47.4	46.5	—	48.3	18.25	.8122
41.4	39.9	—	42.9	23.00	Vitality (VT score) *	39.3	38.2	—	40.3	21.67	**.0214**
69.8	68.1	—	71.6	27.22	Social functioning (SF score)	69.6	68.3	—	71.0	27.15	.8605
64.1	61.5	—	66.7	41.00	Role functioning Emotional (RE score)	63.7	61.7	—	65.7	41.25	.8427
66.3	65.0	—	67.6	20.47	Mental health (MH score)	67.6	66.6	—	68.6	20.39	.1219
RAND-36 Composite Scores:											
57.9	56.6	—	59.2	20.48	Physical Composite Score (PF,RP,BP,GH) ***	54.6	53.5	—	55.6	21.11	**.0001**
60.3	58.9	—	61.8	23.47	Mental Composite Score (VT,SF, RE, MH)	60.0	58.9	—	61.1	22.56	.7404
Percentage (%)					Categorical variables	Percentage (%)					P_in = outpat_
67/33					Gender: female/male	66/34					.4702
16/84					Diabetes (DM): yes/no ***	25/75					**.0000**
39/20/40					DM-Heredity: no/second-/first-degree relative	37/23/40					.6369
76/15/7/2					No tobacco/snuff/smoking/both snuff and smoking	76/14/7/3					.7606
17/83					Snuff: yes/no	17/83					.9944
9/91					Smoking: yes/no	9/91					.7387
21/79					Dropout within one year (yes/no) *	17/83					**.0102**

Boldface indicates statistical significance (*P < .05, **P < .01, ***P < .001).

BMI: Body mass index, BP: Bodily pain, DM: Diabetes mellitus, GH: General health, HDL: High density lipoprotein, LDL: Low density lipoprotein, PF: Physical functioning, RE: Role functioning emotional, RP: Role functioning physical, VT: Vitalty.

**Figure 2.**
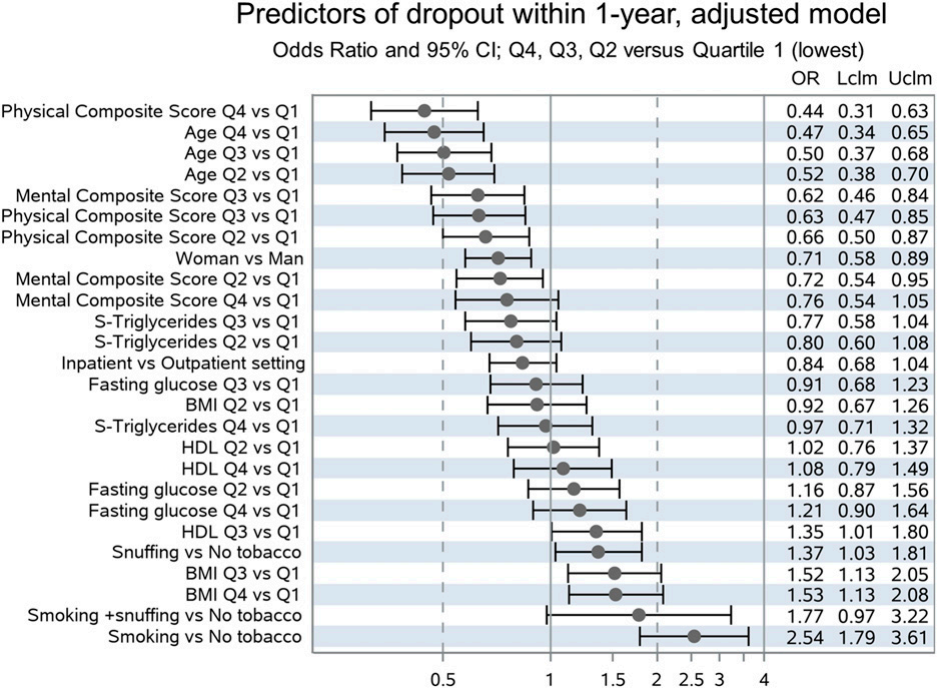
Predictors of dropout within 1-year, adjusted model. Odds Ratio and 95% CI; Q4, Q3, Q2 vs Quartile 1 (lowest).

**Figure 3.**
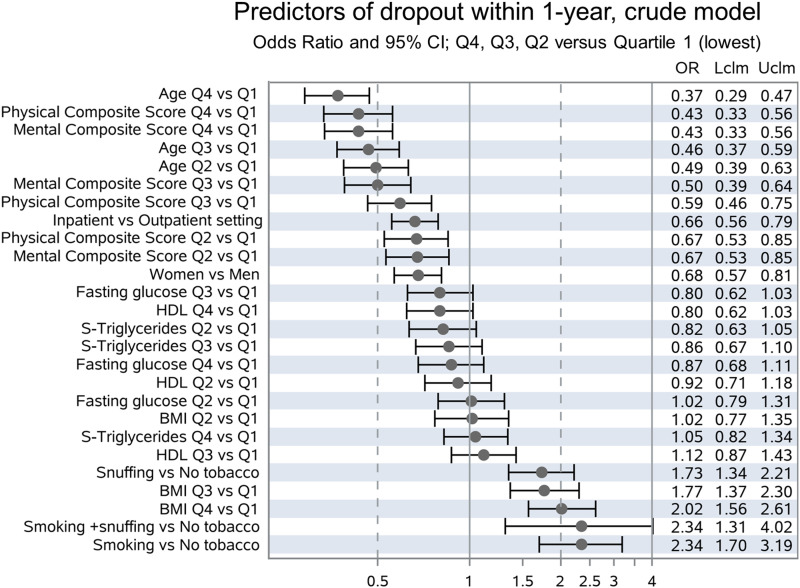
Predictors of dropout within 1-year, crude model. Odds Ratio and 95% CI; Q4, Q3, Q2 vs Quartile 1 (lowest).

Ten baseline variables were chosen by the algorithm as having an impact on the outcome, that is, dropout before 1-year follow-up: Age, Physical Composite Score, Tobacco use, BMI, Sex, Mental Composite Score, Setting, High Density Lipoprotein, Triglyceride and Fasting Glucose. The adjusted model—with odds ratios adjusted for all other included variables—is given in Figure 2: Older age, female sex, and higher composite scores for self-rated physical and emotional health according to RAND-36 are associated with higher rates of full participation in the program. Tobacco use and higher BMI were associated with higher rates of premature dropout. To demonstrate crude Odds ratios, we also built an unadjusted model using the same ten variables (Figure 3). In general, results are similar, but significantly lower risk for premature dropout in the inpatient-setting could only be demonstrated in the unadjusted model.

Patients with incomplete data (Appendix Table 4) have higher rates of premature dropout (29% vs 19%), are younger, have higher BMI, higher rates of smoking and lower composite scores of both physical and emotional functioning.

### Predictor Variables Affected by Treatment Setting

**Figure 4.**
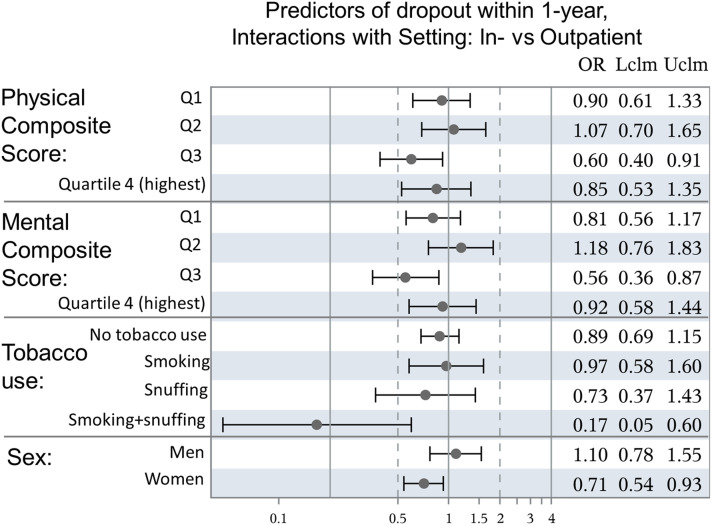
Predictors of dropout within 1-year, Interactions with Setting: In- vs Outpatient.

Four different variables had significant interaction with in- vs outpatient setting (Figure 4): Inpatient-setting was associated with lower risk for premature dropout in women, patients in the third quartiles of Physical and Mental Composite Scores as well as those who were both smoking and snuffing.

## Discussion

The hypothesis that baseline characteristics distinguish patients who fully participate in the intervention from those who drop out prematurely, could be confirmed: older age, higher levels of physical and mental well-being on admission were all associated with lower rates of premature dropout. Higher BMI was associated with higher risk of dropout within one year. Female patients and patients treated in inpatient-setting had lower odds of premature dropout in the crude model, but these associations were attenuated after adjusting for all other variables.

Our second hypothesis, that inpatient-setting is associated with lower rates of premature dropout for patients with lower levels of well-being could not be confirmed: Only patients in the third quartile of physical and mental well-being had higher rates of full participation when treated in inpatient-setting. Women, and patients who were both smoking and snuffing at the start of the intervention had a benefit from inpatient-setting, adherence-wise.

In general, our findings are in line with known predictors of adherence to secondary cardiovascular prevention programs.^22^ That better overall well-being increases adherence to lifestyle interventions, is supported by previous research.^6^ An observational study like the present one cannot establish causality, but the fact that patients who dropped out from the intervention had lower levels of general health on admission makes successful lifestyle-changes an unlikely explanation for early termination of participation. Men being less adherent to the lifestyle intervention program opposes findings in a previous publications.^6^ Reasons behind this divergence cannot be determined fully, but may include differences in culture, treatment recruitment process or other contextual factors. Dropout-rates for this population with metabolic syndrome are higher than those reported for diet-only interventions in patients with type 2 diabetes (0-20%).^23^

The vulnerable groups mentioned above may require other forms of treatment programs than those offered to date. Recent findings supporting sex- and age-specific cardiac rehabilitation^24^ may apply equally to lifestyle interventions for primary cardiometabolic prevention.

For women and tobacco-users, inpatient-setting was associated with higher rates of full participation compared to outpatient-setting. Potential explanations for lower rates of premature dropout from lifestyle interventions in an inpatient-setting are lower degree of decision making required in the inpatient-setting and a higher intensity of intervention enabling patients to achieve measurable change faster. However, these advantages should apply equally—or even especially—to men, which, apart from the small group of users of tobacco both as snuff and cigarettes, could not be confirmed in our study. Attenuation of the adherence-promoting effect of inpatient-setting after adjustment for patient characteristics indicates that differences at group level explain part of that effect. In terms of physical and mental well-being, only patients in the third quartiles benefitted from inpatient-setting. Cutoffs for fourth quartiles of physical and mental composite scores in the present population are close to age standardized means derived from a healthy reference population,^21^ which means that most participants in the respective third quartiles have lower well-being then average of the general background population. Yet, participants with the lowest levels of well-being had similar rates of premature dropout, irrespective of treatment setting. As old age is an established patient-centered barrier to adherence^22^ young age as a risk factor may come as a surprise. However, considering that all participants had elevated cardiometabolic risk (i.e., metabolic syndrome)—being at risk at a younger age is an indicator of increased vulnerability with absence of symptoms, a possibly higher disease-driven barrier to adherence. As the importance of childhood-interventions to change trajectories from cardiovascular risk to manifestation in adulthood is well established^25^ our findings of higher risk of non-adherence in younger adults underlines that interventions may need to be adapted for or specifically targeted at different age groups.

Overall, the more vulnerable the participant, the more likely was he/she to drop out prematurely. Clinically, we may conclude that additional support for the most vulnerable patients should be explored as a viable way to improve adherence.

### Study Strengths and Limitations

The presented data are unique in coming from patients participating in similar intensive multimodal lifestyle interventions implemented in general health care in two different settings. Compared to results from protocols developed exclusively for research purposes (proof of concept), data from the present study are thus directly applicable to an existing treatment program. To our knowledge, this is also one of the first studies which focuses on adherence to an intensive multimodal lifestyle intervention. The common approach of focusing on outcomes and managing non-adherence as a modulating factor is, of course fully justified. However, unlike most other treatments, effects of lifestyle interventions are 100% dependent on patient cooperation and adherence. Therefore, establishing who will be able to follow through with an intervention may be seen as having high priority.

Several limitations must be acknowledged: As in most real-world studies of adherence, less is known about patients with incomplete data. As a group, these patients have higher rates of premature dropout. As they also have baseline characteristics that are associated with premature dropout in those with complete data, their exclusion is likely to have attenuated our findings. Dropout-rates among outpatients with incomplete data were almost twice as high, indicating that estimates of the participation-improving effect of inpatient-setting in this study are conservative. Another real-world limitation is that decision on treatment setting was based on geography and effects ascribed to setting may be confounded by where patients live. However, this study is an important first step to further explore the effect of treatment setting. Data collected at baseline are insufficient to give a clear profile of actual living habits, that is, what patients eat, how physically active they are, their sleep hygiene and stress management techniques. As these habits are the primary targets of the intervention such a lifestyle-profile is not only an important complement to risk factors such as BMI, blood pressure and lipid profile but also a potentially important patient characteristic to consider when deciding upon an intervention. Actual living habits have been described as downstream determinants of chronic disease etiology, with interpersonal relations such as peer pressure and general society as mid- and up-stream determinants, respectively.^26^ We have no data on these mid- and up-stream determinants of behavior, although they are likely to influence not only an individual’s living habits, but also his/her ability to comply with lifestyle interventions. Changes in medication and important life events like employment issues, family issues and financial issues may determine a patient’s decision to discontinue participation in the intervention, thus limiting the predictive value of baseline characteristics. Finally, for lifestyle interventions to be effective, delivery must be adapted to local context (health care system, socio-economic setting, legal framework, etc.) which—together with the delay in time between data collection and analysis—limits external validity of findings from a particular setting.

## Conclusions

The less favorable a patient’s baseline characteristics, the higher the risk of premature dropout, that is, those patients who need it most are also most likely to drop out from the lifestyle intervention. Women, tobacco-users, and participants with levels of well-being that are only slightly below average have higher rates of full participation when treated in inpatient-setting. Men, younger patients, patients with lowest levels of physical and mental well-being as well as patients with higher BMI are especially prone to premature dropout independent of treatment setting. Further research is needed to establish whether other approaches, such as taking patients preferences into account to a greater extent and offering individualized programs as a complement to group-based ones, could improve rates of full participation in multimodal lifestyle interventions for those most vulnerable groups.

## Supplemental Material

Supplemental Material - Intensive Lifestyle Intervention for Cardiometabolic Prevention Implemented in Healthcare: Higher Risk Predicts Premature Dropout Supplemental Material for Intensive Lifestyle Intervention for Cardiometabolic Prevention Implemented in Healthcare: Higher Risk Predicts Premature Dropout in Benno Krachler, MD, PhD, Anna Söderholm, PhD, Fanny Ekman, MD, Frida Lindberg, MD, Joakim Lindbäck, MD, Johan Nilsson Sommar, PhD, Eva-Lotta Glader, MD, PhD, and Bernt Lindahl, MD, PhD in American Journal of Lifestyle Medicine

Supplemental Material - Intensive Lifestyle Intervention for Cardiometabolic Prevention Implemented in Healthcare: Higher Risk Predicts Premature Dropout Supplemental Material for Intensive Lifestyle Intervention for Cardiometabolic Prevention Implemented in Healthcare: Higher Risk Predicts Premature Dropout in Benno Krachler, MD, PhD, Anna Söderholm, PhD, Fanny Ekman, MD, Frida Lindberg, MD, Joakim Lindbäck, MD, Johan Nilsson Sommar, PhD, Eva-Lotta Glader, MD, PhD, and Bernt Lindahl, MD, PhD in American Journal of Lifestyle Medicine

Supplemental Material - Intensive Lifestyle Intervention for Cardiometabolic Prevention Implemented in Healthcare: Higher Risk Predicts Premature Dropout Supplemental Material for Intensive Lifestyle Intervention for Cardiometabolic Prevention Implemented in Healthcare: Higher Risk Predicts Premature Dropout in Benno Krachler, MD, PhD, Anna Söderholm, PhD, Fanny Ekman, MD, Frida Lindberg, MD, Joakim Lindbäck, MD, Johan Nilsson Sommar, PhD, Eva-Lotta Glader, MD, PhD, and Bernt Lindahl, MD, PhD in American Journal of Lifestyle Medicine

Supplemental Material - Intensive Lifestyle Intervention for Cardiometabolic Prevention Implemented in Healthcare: Higher Risk Predicts Premature Dropout Supplemental Material for Intensive Lifestyle Intervention for Cardiometabolic Prevention Implemented in Healthcare: Higher Risk Predicts Premature Dropout in Benno Krachler, MD, PhD, Anna Söderholm, PhD, Fanny Ekman, MD, Frida Lindberg, MD, Joakim Lindbäck, MD, Johan Nilsson Sommar, PhD, Eva-Lotta Glader, MD, PhD, and Bernt Lindahl, MD, PhD in American Journal of Lifestyle Medicine

## Data Availability

Anonymous and limited data can be made available on request. The data underlying this article will be shared upon reasonable request to the corresponding author.

## Appendix

### Glossary

BMI: Body mass index
BP: Bodily pain
DM: Diabetes mellitus
GH: General health
HDL: High density lipoprotein
LDL: Low density lipoprotein
PF: Physical functioning
RE: Role functioning emotional
RP: Role functioning physical
VT: Vitality
